# Rituximab and Fibrillary Glomerulonephritis: Interest of B Cell Reconstitution Monitoring

**DOI:** 10.3390/jcm7110430

**Published:** 2018-11-09

**Authors:** Claire Leibler, Anissa Moktefi, Marie Matignon, Céline Debiais-Delpech, Julie Oniszczuk, Dil Sahali, José L. Cohen, Philippe Grimbert, Vincent Audard

**Affiliations:** 1 AP-HP (Assistance Publique-Hôpitaux de Paris), Service de Néphrologie et Transplantation, Centre de Référence Maladie Rare Syndrome Néphrotique Idiopathique, Hôpital Henri-Mondor/Albert-Chenevier, F-94000 Créteil, France; claire.leibler@yahoo.fr (C.L.); marie.matignon@aphp.fr (M.M.); julieon@free.fr (J.O.); dil.sahali@inserm.fr (D.S.); philippe.grimbert@aphp.fr (P.G.); vincent.audard@aphp.fr (V.A.); 2 Université Paris-Est, UMR-S955, UPEC, F-94000 Créteil, France; jose.cohen@inserm.fr; 3 Inserm, U955, Equipe 21, F-94000 Créteil, France; 4 AP-HP, Département de Pathologie, Hôpital Henri-Mondor/Albert-Chenevier, F-94000 Créteil, France; 5 Centre de Référence des Amyloses Primitives et des Maladies de Dépôts d’Immunoglobulines Monoclonales, F-86000 Poitiers, France; celine.debiais@hotmail.fr; 6 Département de Pathologie, Centre Hospitalier Universitaire de Poitiers, F-86000 Poitiers, France; 7 APHP, Hôpital Henri Mondor-A. Chenevier, Centre d’Investigation Clinique Biothérapie, F-94000 Créteil, France

**Keywords:** fibrillary glomerulonephritis, rituximab, B cell reconstitution

## Abstract

Fibrillary glomerulonephritis (FGN) is a rare glomerular disease characterized by glomerular deposition of randomly arranged non-amyloid fibrils. FGN has a poor renal prognosis and its optimal treatment is a medical challenge. Rituximab therapy has recently emerged as a promising approach even though its mechanism of action remains hypothetical. We describe the case of a 55-year-old woman with FGN successfully treated by rituximab. During the 36-month follow-up, she had three relapses of FGN, occurring each time in the context of B cell recovery. Investigation of the distribution of B cell subpopulations at the time of the third relapse showed, as previously described for some immunological diseases, an increase in the proportion of switched memory B cells relative to healthy subjects, whereas global memory B cell pool was not yet recovered. This case suggests that B cell reconstitution should be carefully monitored in the management of FGN treated with rituximab.

## 1. Introduction

Fibrillary Glomerulonephritis (FGN) is a rare glomerular disease characterized by the deposition of randomly distributed non-amyloid fibrils in the mesangium and capillary basement membrane of the glomeruli [1]. On immunofluorescence, the glomeruli deposits are typically composed of polyclonal immunoglobulin G (IgG) and contain predominantly IgG4 [2,3]. FGN is a severe renal disease with a poor renal prognosis [2,3,4]. The pathophysiological processes involved in the occurrence of FGN remain unknown, but DnaJ homolog subfamily B member 9 (DNAJB9) staining on glomerular deposits has recently emerged as a specific immunohistochemical marker for FGN [5,6,7]. In most uncontrolled case-series, the use of immunosuppressive agents did not demonstrate a clear benefit concerning renal outcome. Recent findings suggest that rituximab therapy may slow the progression of chronic kidney disease, but the underlying mechanisms supporting its efficacy remain to be determined [3,8,9,10]. This therapeutic approach has not been validated in large controlled studies, and the close temporal relationship between B lymphocyte reconstitution and FGN relapse is yet to be determined. Here, we present a patient with biopsy-proven FGN who exhibited three relapses of nephrotic syndrome during 36 months of follow-up (8, 16, and 25 months after the first rituximab administration), occurring each time in the context of B lymphocyte recovery. This finding led us to investigate B cell subpopulation distribution at the time of the third relapse.

## 2. Case Presentation

A 55-year-old woman who had a medical history of hypertension treated with a calcium blocker (amlodipine 5 mg/d) and an angiotensin converting enzyme (ACE) inhibitor (perindopril 5 mg/d) was referred to our nephrology department for the investigation of a proteinuria (10 g/day). Physical examination was unremarkable, with a blood pressure of 125/75 mmHg. Laboratory investigations showed a nephrotic syndrome associated with microscopic hematuria at 10^5^/mm^3^, without renal failure (creatinine level of 80 µmol/L, eGFR (estimated glomerular filtration rate) of 69 mL/min/1.73 m^2^ according to the MDRD (Modification of Diet in Renal Disease) formula). Serum protein electrophoresis showed low gamma globulin levels of 4 g/L (normal range 8–12 g/L) associated with a monoclonal IgM kappa spike (<1 g/L). Urine protein electrophoresis did not detect Bence Jones proteinuria, and the serum kappa/lambda free light chain ratio assessed by immunonephelometric assays was within the normal range (ratio: 0.7; normal range: 0.7–1.56).

**Figure 1:**
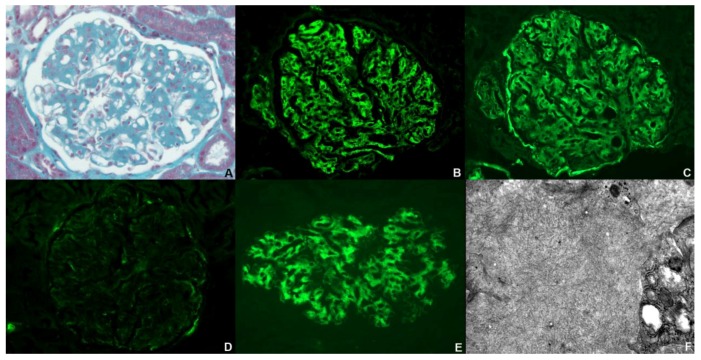
Representative kidney biopsy findings showing FGN. (**A**) Marked mesangial matrix expansion and focal thickening of glomerular basement membrane by deposits (Masson’s trichrome staining × 400); immunofluorescence: mesangial and capillary loop smudgy positivity for IgG (**B**) and C3 (**C**) (×400); (**D**) weak capillary wall staining for IgM (×400); (**E**) immunofluorescence staining for IgG subclasses revealed positive staining for IgG4 (×400); (**F**) electron microscopy: deposits were composed of randomly arranged non branching fibrils of 16 nm in diameter (×40,000).

A percutaneous renal biopsy was performed in April 2013 (description of detailed methods in Appendix A). The renal biopsy specimen consisted of renal cortex with 14 glomeruli including two obsolescent glomeruli. Other glomeruli exhibited diffuse mesangial expansion and focal thickening of glomerular basement membrane (Figure 1A). Congo-Red staining was negative and the tubulointerstitial areas showed mild interstitial fibrosis (<10%). Immunofluorescence studies demonstrated intense mesangial and capillary loop smudgy staining for IgG (3+) (Figure 1B), associated with glomerular deposition of complement component 3 (C3) (3+) (Figure 1C). Immunofluorescence revealed positive staining for both kappa and lambda light chains. We observed weak capillary wall staining for IgM (Figure 1D). Analysis of the glomerular IgG subclass distribution demonstrated positive staining for IgG4, and was negative for other IgG subclasses (Figure 1E). Electron microscopy showed typical features of FGN, i.e., the presence of diffuse mesangial infiltration by randomly oriented fibrils with a diameter of 16 nm (Figure 1F).

Bone marrow examination revealed no tumoral infiltration and positron emission tomography-computed tomography (PET-CT) did not detect highly hypermetabolic lesions.

**Figure 2:**
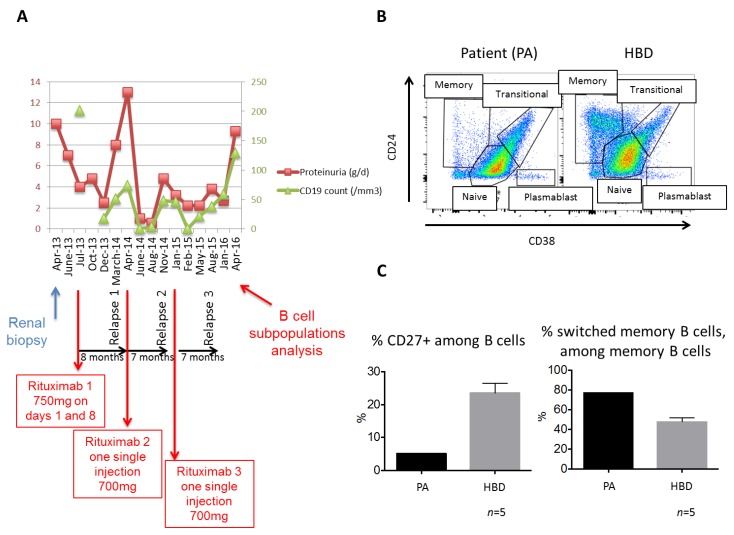
Close temporal relationship between CD19+ cell counts and proteinuria relapse during follow-up and analysis of B cell subgroups. (**A**) Increased proteinuria was strongly associated with increased CD19+ cell counts after each rituximab infusion. Note that each relapse occurred approximately seven to eight months after each administration of rituximab; (**B**) characterization of B cell subgroups (transitional, naive, and memory B cells, and plasmablasts) according to CD24 and CD38 markers, among CD19 gated cells; (**C**) representative dot plots from the patient (PA) and healthy blood donors (HBD) (*n* = 5). The patient displayed a lower proportion of CD27+ B cells among CD19+ cells but a higher proportion of switched memory B cells (among CD19+CD27+) relative to HBD (*n* = 5).

Proteinuria decreased from 10 g/day to 7.3 g/day after increasing the dose of ACE inhibitor in April 2013, however proteinuria remained in a nephrotic range and albuminemia levels were unchanged (25 g/L). In July 2013, the patient received two doses of rituximab intravenously (375 mg/m^2^), separated by one week. Five months after rituximab administration, she achieved partial remission with a decrease of proteinuria levels > 50% (2.5 g/day) (Figure 2A). However, eight months after the first administration of rituximab, relapse of nephrotic syndrome (proteinuria 12.8 g/day) occurred, which was successfully treated by a single infusion of rituximab (375 mg/m^2^). Two new relapses of nephrotic syndrome occurred during the follow-up and were each successfully treated by a single infusion of rituximab (administered 15 days and one month, respectively, after confirmation of FGN relapse) (Figure 2A). Each relapse occurred between seven and eight months after the previous rituximab administration. Renal function was stable (eGFR = 68 mL/min/1.73 m^2^) at the 36-month follow-up. Strikingly, each relapse occurred in the context of B cell recovery (51, 48, and 38/mm^3^) (Figure 2A).

We next analyzed the distribution of circulating B cell subsets at the time of the third relapse (15 months after the last rituximab infusion) by flow cytometry and compared our results to those observed in healthy blood donors (HBD) (description of detailed methods in Appendix A, Table A1; gating strategy used to study B cell subpopulations are shown in supplementary materials). The CD19+ count was within the normal range (133/mm^3^), however the distribution of B cell subpopulations was different from that of HBD (Figure 2B). The proportion of transitional (immature) B cells (CD19+CD24hiCD38hi) was higher in our patient (20.8%) than in HBD (mean ± SEM: 9.7 ± 1.6%). Moreover, the proportion of memory B cells (CD19+CD27+) among CD19+ cells was much lower in our patient (5%) than in HBD (mean ± SEM: 28.3 ± 5%). Among memory CD19+CD27+ B cells, the proportion of switched memory B cells was higher than HBD (Figure 2C), whereas proportion of plasmablasts (0.6%) was in the same range as HBD (mean ± SEM: 0.42 ± 0.05%).

## 3. Discussion

Since the pathogenesis of FGN remains uncertain, optimal treatments are still challenging and controversial. Cytotoxic drugs were considered initially, but failed to show a clear benefit on kidney disease outcome [2,4,8]. Rituximab therapy has recently emerged as a promising alternative therapeutic option to halt the progression of renal disease [8,9,10]. A recent study from the University of Northern Carolina showed that of nine rituximab-treated patients, only 11% achieved a stable condition, however those treated earlier and those who had less sclerosis at the time of biopsy responded better to rituximab treatment [11]. Due to missing data concerning optimal dose of rituximab to treat FGN, our initial protocol (two doses of 375 mg/m^2^ separated by one week) was largely extrapolated from the use of rituximab in other glomerular diseases [12,13]. Mechanisms underlying its efficacy in the context of FGN have not been elucidated, and the potential relationship between B cell reconstitution and proteinuria relapse remains to be clearly demonstrated. In the study of Hogan et al., the B cell counts after rituximab administration were not systematically monitored [9]. One patient described by Collins et al. displayed an increase in proteinuria level in the context of B cell recovery [10] and Javaugue et al. reported a patient with relapse of FGN one year after rituximab therapy with reappearance of detectable CD19+ cells [8]. Our patient experienced three relapses of proteinuria concomitant with B cell recovery. For each relapse, the proteinuria level decreased substantially after one additional single infusion of rituximab. This finding led us to analyze B cell subgroup distributions at the time of the third relapse. The patient’s global B cell subgroup distribution differed greatly from that of HBD. The patient had a higher proportion of transitional immature B cells, associated with a very low proportion of memory B cells. This alteration of B cell subgroup partitioning may be related to B cell reconstitution after rituximab administration. Indeed, our phenotypic study was performed after three courses of rituximab, 15 months after the last infusion. The timing of B cell reconstitution after rituximab treatment, defined by global CD19+ counts, varies from nine to 20 months, depending on the pathological context [14,15]. Delayed memory B cell recovery after rituximab therapy, as observed in our patient, has been previously described in various autoimmune settings [14,15,16]. In these clinical settings, immunological disease activity appears to be dependent on the timing of the reappearance of specific B cell subpopulations. Colluci et al. showed that the recovery of switched memory B cells at nine months was a strong risk factor for subsequent idiopathic nephrotic syndrome relapses [17]. Notably, our patient displayed a higher proportion of switched memory B cells than HBD when her global memory B cell pool was not completely recovered. Rituximab is a depleting chimeric antibody directed against B cells but it does not directly affect mature plasma cells, the effector B cells responsible for the secretion of IgG, as they do not express the surface molecule CD20 [18]. Hence, the efficacy of rituximab in FGN cannot be directly related to a decreased ability of plasma cells to secrete pathogenic IgG4. Thus, we hypothesize that B cells in the context of FGN, as in other autoimmune disease such as lupus, are playing a major role as antigen presenting cells and stimulators of effector T cell populations, as well as forming germinal centers. In this regard, we believe that the switched memory B cells may be a predominant mediator of this process. Thus, the clinical efficacy of rituximab may be related to effective depletion of switched memory B cells. Notably, the proportion of circulating plasmablasts did not seem to be modified.

## 4. Conclusions

These data concerning a rare glomerular disease and based on a unique case emphasize the need to closely monitor B cell reconstitution after rituximab therapy in FGN patients. A large number of prospectively enrolled patients are needed to clearly demonstrate that rituximab administration may be considered as soon as CD19+ cells become detectable, despite the absence of clinically evident relapse. Because extensive B lymphocyte subpopulation analysis is not yet routinely performed in clinical practice, further studies are required to determine the accurate role of switched memory B cells in the pathogenesis of FGN.

## Supplementary Materials

The following are available online at http://www.mdpi.com/2077-0383/7/11/430/s1, Figure S1: Gating strategy used to study B cell subpopulations in our patient (A) and in one representative healthy blood donor (B). Figure S2: Representative FACS plots showing (A) patient’s unstained peripheral blood mononuclear cells (PBMC); (B) patient’s PBMC stained for CD19 only; (C): patient’s B cell subpopulations.

## Appendix A. Detailed Methods

### Appendix A1. Analysis of Renal Biopsy Specimen

The patient’s biopsy specimen was prepared for light, immunofluorescence, and electron microscopy by standard techniques. For immunofluorescence (IF), 3 µm cryostat sections were incubated with polyclonal fluorescein isothiocyanate-conjugated (FITC) antibodies to human γ, α, and µ Ig heavy chains; κ and λ light chains; C1q; C3; fibrinogen; and albumin (Dako, Glostrup, Denmark). For detection of IgG subclasses, cryosections of the renal biopsy specimen were stained using monoclonal antibodies specific for IgG1, IgG2, IgG3, and IgG4 (Sigma-Aldrich; Saint Louis, MO, USA; 1 in 30 dilution). IF staining intensity was graded on a scale of 0 to 3+ (0 = absence, 1+ = mild, 2+ = moderate, 3+ = intense) by one observer (AM). Ultrastructural analysis was performed using a JEM 1010 electron microscope (JEOL, Tokyo, Japan).

### Appendix A2. B Cell Subset Analysis by Flow Cytometry

Human peripheral blood mononuclear cells (PBMCs) were isolated through a Ficoll/Hypaque density gradient (Eurobio, France). Five healthy subjects (healthy blood donors (HBD)) from the French Blood Establishment were included as controls. PBMCs were freshly stained at 4 °C for 20 min with fluorochrome-conjugated monoclonal antibodies to characterize B cell subsets by using the following reagents: CD19 (clone HIB19), CD24 (ML5), CD38 (HB-7), and CD27 (1A4CD27) from Beckman and Coulter; IgD (IA-62) and IgM (SA-DA4) from Ebiosciences (San Diego, CA, USA). B cell subpopulation analyses according to their surface markers are summarized in Table A1.

The dot plots demonstrating the gating strategy used to study B cell subpopulations among blood lymphocytes of (A) the patient and (B) one representative healthy blood donor are shown in Supplemental Figure S1.

Control antibody stains were performed for both the patient and the healthy blood donors to confirm gating strategies. This included unstained PBMCs and PBMCs stained with CD19 only. Dot plots are shown in Supplemental Figure S2.

Cells were analyzed using a FACS CANTO (BD Bioscience, Franklin Lakes, NJ, USA) and FlowJo v.10 software (Treestar, Flowjo LLC, Ashland, OR, USA).

The study was performed in accordance with the ethical standards of the Helsinki declaration, and has been approved by our local institutional review board: IRB 412 Mondor N° 00003835 and by the Comité de Protection des Personnes d’Ile de France IV (N° 2016/25NICB).

Written consent was obtained from the patient to perform B cell subpopulation analysis.

**Table A1:** Characteristics of circulating B cell subpopulations.

Circulating B Cell Subpopulations	Markers
Transitional (Immature)	CD19+CD24^hi^CD38^hi^
Antigen independent maturation	
Mature naïve	CD19+CD24^int^CD38^int^
Antigen dependent maturation in lymphoid follicles: TFH-B cells cooperation	
Memory	CD19+CD27+ or CD19+CD24^hi^CD38−
Switched memory	CD19+CD27+IgD−
Unswitched memory	CD19+CD27+IgD+
Plasma cells	CD19+CD24−CD38^hi^
