# Supplementary material for: Rituximab and Fibrillary Glomerulonephritis: Interest of B Cell Reconstitution Monitoring
Source: J Clin Med. 2018 Nov 9;7(11):430. doi: 10.3390/jcm7110430 (PMC6262590; doi:10.3390/jcm7110430)
Supplement: Supplementary file 1 [file jcm-07-00430-s001.zip › supplementary/Leibler et al Fig S1 proof.pptx]

## Slide 1
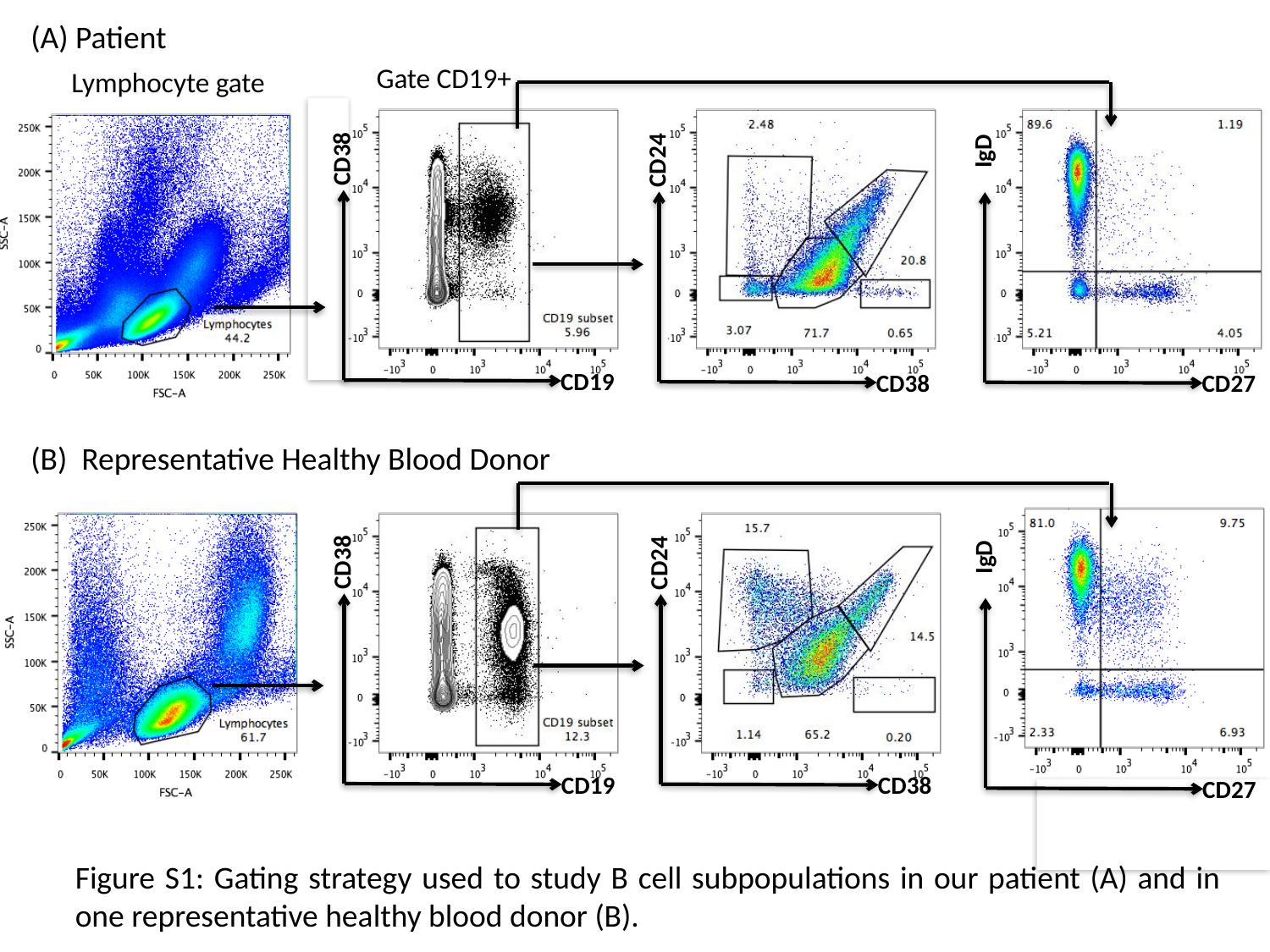

(A) Patient
Gate CD19+
Lymphocyte gate
CD38
CD24
IgD
CD19
CD38
CD27
(B) Representative Healthy Blood Donor
CD38
CD24
IgD
CD19
CD38
CD27
Figure S1: Gating strategy used to study B cell subpopulations in our patient (A) and in one representative healthy blood donor (B).
