# Supplementary material for: Rituximab and Fibrillary Glomerulonephritis: Interest of B Cell Reconstitution Monitoring
Source: J Clin Med. 2018 Nov 9;7(11):430. doi: 10.3390/jcm7110430 (PMC6262590; doi:10.3390/jcm7110430)
Supplement: Supplementary file 1 [file jcm-07-00430-s001.zip › supplementary/Leibler et al Fig S2 proof.pdf]

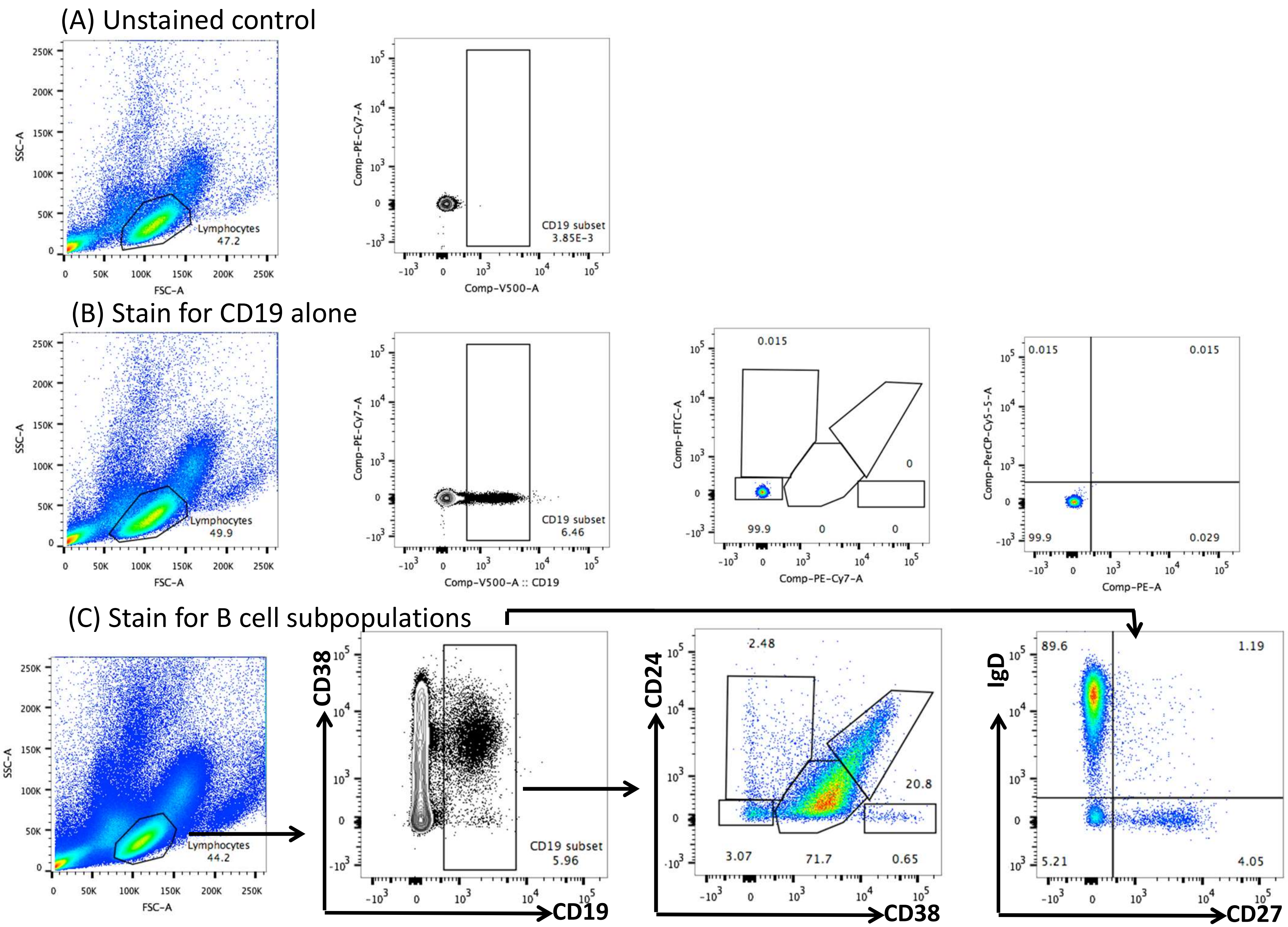

Figure S2: Representative FACS plots showing (A) patient's unstained peripheral blood mononuclear cells (PBMC); (B) patient's PBMC stained for CD19 only; (C): patient's B cell subpopulations.
