# Supplementary material for: Rituximab and Fibrillary Glomerulonephritis: Interest of B Cell Reconstitution Monitoring
Source: J Clin Med. 2018 Nov 9;7(11):430. doi: 10.3390/jcm7110430 (PMC6262590; doi:10.3390/jcm7110430)
Supplement: Supplementary file 1 [file jcm-07-00430-s001.zip › supplementary/Leibler et al, Fig S2 proof.pptx]

## Slide 1
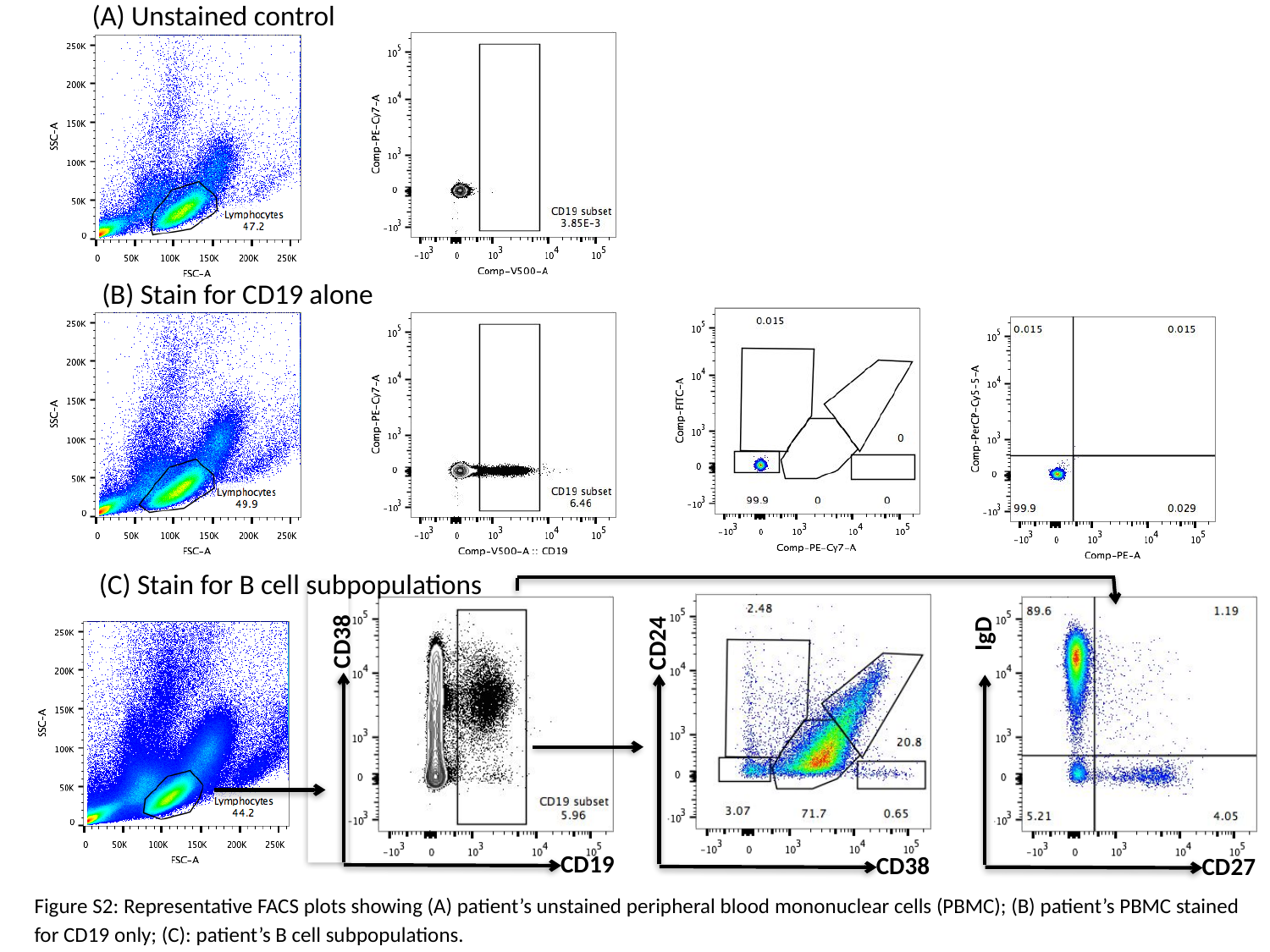

(A) Unstained control
(B) Stain for CD19 alone
(C) Stain for B cell subpopulations
CD38
CD24
IgD
CD19
CD38
CD27
Figure S2: Representative FACS plots showing (A) patient’s unstained peripheral blood mononuclear cells (PBMC); (B) patient’s PBMC stained for CD19 only; (C): patient’s B cell subpopulations.
